# Crossover from lattice to plasmonic polarons of a spin-polarised electron gas in ferromagnetic EuO

**DOI:** 10.1038/s41467-018-04749-w

**Published:** 2018-06-13

**Authors:** J. M. Riley, F. Caruso, C. Verdi, L. B. Duffy, M. D. Watson, L. Bawden, K. Volckaert, G. van der Laan, T. Hesjedal, M. Hoesch, F. Giustino, P. D. C. King

**Affiliations:** 1 SUPA, School of Physics and Astronomy, University of St. Andrews, St. Andrews, KY16 9SS UK; 2 Diamond Light Source, Harwell Campus, Didcot, OX11 0DE UK; 3 Department of Materials, University of Oxford, Parks Road, Oxford, OX1 3PH UK; 4 Institut für Physik and IRIS Adlershof, Humboldt-Universität zu Berlin, Berlin, 12489 Germany; 5 Department of Physics, University of Oxford, Oxford, OX1 3PU UK; 6 ISIS, STFC, Rutherford Appleton Laboratory, Didcot, OX11 0QX UK; 7 DESY Photon Science, Deutsches Elektronen-Synchrotron, Hamburg, D-22603 Germany; 8 Department of Materials Science and Engineering, Cornell University, Ithaca, New York, 14853 USA

## Abstract

Strong many-body interactions in solids yield a host of fascinating and potentially useful physical properties. Here, from angle-resolved photoemission experiments and ab initio many-body calculations, we demonstrate how a strong coupling of conduction electrons with collective plasmon excitations of their own Fermi sea leads to the formation of plasmonic polarons in the doped ferromagnetic semiconductor EuO. We observe how these exhibit a significant tunability with charge carrier doping, leading to a polaronic liquid that is qualitatively distinct from its more conventional lattice-dominated analogue. Our study thus suggests powerful opportunities for tailoring quantum many-body interactions in solids via dilute charge carrier doping.

## Abstract (web summary)

Many-body interactions in solids offer opportunities to realize striking physical properties. Here the authors demonstrate the formation of plasmonic polarons in Eu_1-*x*_Gd_*x*_O and their tunability with charge carrier doping, providing a route to tailoring quantum many-body interactions in solid.

## Introduction

A pronounced electron−phonon coupling in solids is known to mediate the formation of polarons—composite quasiparticles of an electron dressed with a phonon cloud^1^. Polarons exhibit significantly enhanced quasiparticle masses that modify charge carrier transport and are proposed to play a key role in unconventional superconducting and colossal magnetoresistive states in compounds including high-*T*_c_ cuprates^2,3^, SrTiO_**3**_-based electron gases^4–8^, manganites^9,10^ and superconducting monolayer FeSe^11^. Developing control over polaronic states in solids therefore holds exciting potential for manipulating the collective states of quantum materials. Phonons, however, are typically only weakly modified for experimentally accessible tuning parameters. In contrast, we demonstrate in this work that polarons which are formed via a coupling to collective plasmon excitations of an electron gas provide a highly tuneable system.

**Fig. 1:**
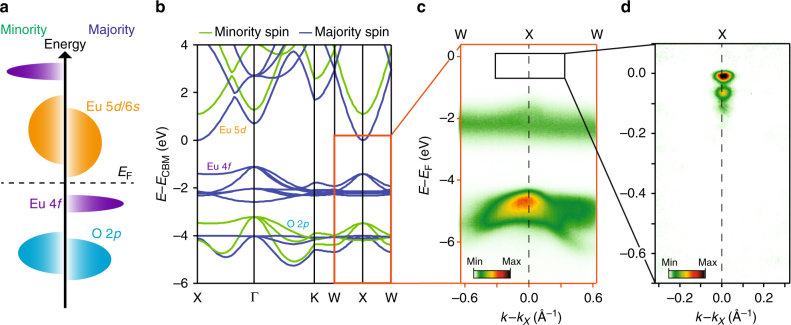
Electronic structure of the ferromagnetic semiconductor EuO. **a** Schematic energy level diagram of EuO, with a half-filled Eu 4*f* band split by strong Coulomb interactions yielding a band gap between Eu 4*f* (5*d*) valence (conduction) bands. **b** DFT calculations of the electronic structure reproduce these general features and indicate the conduction band minimum (CBM) is located at the Brillouin zone face, X, point. **c** ARPES measurements ($h\nu$ = 48 eV) from a lightly Gd-doped sample (Eu_1−*x*_Gd_*x*_O, *x* = 0.023) qualitatively match the DFT valence band dispersions. **d** The charge carrier doping additionally populates the spin-majority Eu 5*d* conduction-band state at the X-point (region shown by black box in (**c**))

We investigate this in the doped ferromagnet EuO. Stoichiometric EuO is insulating, with a Curie temperature of *T*_c_ = 69 K. In an ionic picture, Eu^2+^ has seven electrons half-filling the Eu 4*f* shell. These unpaired electrons align according to Hund’s rule, producing a large energetic splitting between occupied and unoccupied Eu 4*f* states via strong onsite Coulomb interactions, *U* (Fig. 1a). The temperature-dependent magnetisation follows an almost perfect Brillouin function^12^, reaching a saturation magnetisation of 7 *μ*_B_/Eu. EuO is thus often described as an almost ideal manifestation of a Heisenberg ferromagnet^13^. Oxygen reduction or atomic substitution of trivalent ions, for example Gd^3+^, for divalent Eu^2+^ dopes electrons into an Eu-derived 5*d* conduction band. This has a dramatic effect on its physical properties, increasing *T*_c_^14^, stabilising a giant temperature-dependent metal−insulator transition with up to 13 orders of magnitude change in conductivity^15^, inducing giant magnetoresistance^16^, and realising a half-metallic phase, enabling almost 100% spin injection into Si and GaN^17^.

We synthesise epitaxial films of Eu_1−*x*_Gd_*x*_O via oxide molecular-beam epitaxy (MBE), allowing fine control over its charge carrier concentration, and transfer these in situ to a synchrotron-based angle-resolved photoemission spectroscopy (ARPES) system (see Methods). This provides a powerful opportunity to probe the electronic structure of this three-dimensional and air-sensitive compound. Our corresponding ARPES measurements, together with ab initio many-body calculations^18,19^, reveal how charge carrier doping fundamentally changes the nature of the underlying electronic liquid in EuO. In particular, we directly image the emergence of well-defined satellites in the spectral function whose energy separation, unusually, grows as $\sqrt n$, where *n* is the free carrier density. This points to an intriguing polaronic state, arising due to the coupling of the induced charge carriers to the conduction-electron plasmon excitations.

## Results

### Electronic structure of lightly doped EuO

**Fig. 2:**
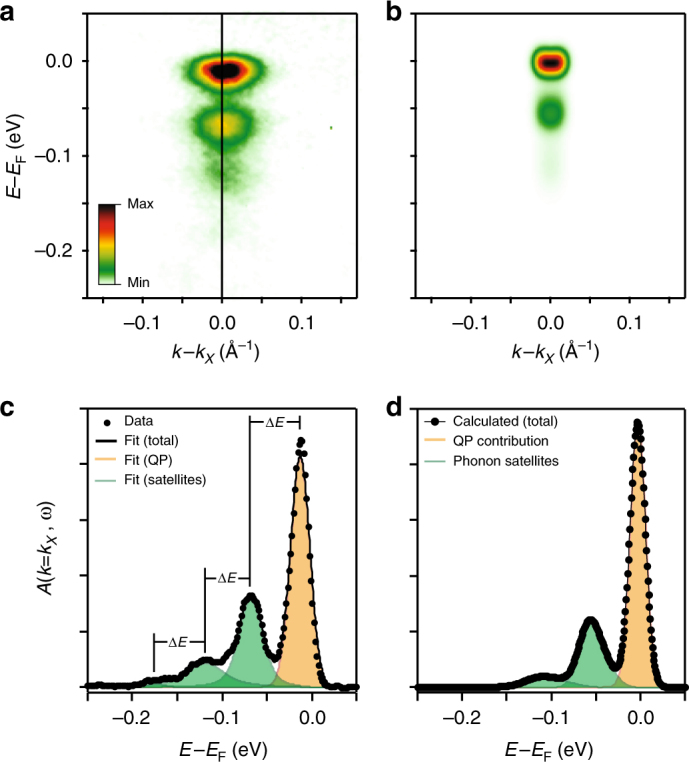
Spectroscopic observation of lattice polarons in dilutely doped EuO. **a** Measured and **b** calculated occupied part of the single-particle spectral function of dilutely doped Eu_1−*x*_Gd_*x*_O (*x* = 0.023, *n* = 9.3×10^17^ cm^−3^, see Methods). Replica satellite bands below the main quasiparticle band that crosses the Fermi level are evident. **c** These are visible up to third order in an EDC taken at *k* = *k*_X_, visible as distinct peaks (green shading) separated from the main quasiparticle peak (orange shading) by integer multiples of the LO phonon energy. **d** Similar features are evident in our ab initio many-body calculations which explicitly treat electron−phonon coupling from first principles

Figure 1 summarises the electronic structure of our Gd-doped EuO films. After incorporating an onsite Coulomb repulsion, density-functional theory calculations (DFT, see Methods) reproduce the generic features of the electronic structure described above, including its ferromagnetic nature. A majority-spin Eu 4*f* level is fully occupied, sitting above an O 2*p*-derived valence band that inherits a much weaker exchange splitting. Our ARPES measurements of the valence band dispersions from a lightly doped sample (*x* = 0.023, Fig. 1c) are in good general agreement with these DFT calculations, as well as with prior ARPES studies of the valence band electronic structure of EuO^20,21^. For a weakly interacting semiconductor, charge carrier doping would simply induce a rigid shift of the Fermi level into the majority-spin Eu 5*d* conduction band, whose minimum is located at the X-point of the Brillouin zone (see also Supplementary Fig. 1). Consistent with previous experiment^21^, we indeed find that conduction-band states become populated at X with electron doping (Fig. 1d). Our measured spectra (evident in Fig. 1d and shown magnified in Fig. 2a), however, are not consistent with a simple rigid-band filling.

Instead, we observe a series of replica bands offset in energy from the main quasiparticle band which intersects the Fermi level. This is a characteristic spectroscopic signature of Fröhlich polaron formation^22,23^, whereby strong electron−phonon interactions give rise to shake-off excitations involving small **q** scattering processes. These yield satellite features in the spectral function, shifted to successively higher binding energy and evenly spaced by the corresponding phonon mode frequency. From our measured energy-distribution curves (EDCs, Fig. 2c), we observe three such distinct replica bands which, from fitted peak positions, are separated by a constant value of $\Delta E \approx (56\,\pm\, 3)\,{\mathrm {meV}}$. This agrees well with the longitudinal optical phonon mode frequency measured from EuO single crystals^24^ as well as with the optical phonon branch obtained from our ab initio calculations (Supplementary Fig. 2). We thus attribute these observed spectral features in very lightly doped EuO as polaronic satellites arising from a strong electron−phonon coupling.

To confirm that this is an intrinsic property of the spectral function, we perform many-body ab initio calculations within the cumulant expansion method^25,26^, thereby including the effects of electron−phonon coupling from first principles (see Methods). Apart from a small overall energetic shift, our calculations, performed for the same carrier doping as in our experiments, yield a spectral function in excellent agreement with the one measured by ARPES (Fig. 2c, d), including the spacing and approximate spectral weights of the replica features. Indeed, this level of agreement is remarkable given that the calculations are performed fully ab initio and there are no tuning parameters employed. They reveal a pronounced quasiparticle mass renormalisation, m^*^/*m*_0_ = 2.1, where *m*_0_ is the bare band mass, pointing to a strong electron−phonon coupling, and supporting that dilutely doped EuO is in the polaronic limit. We note that similar spectral features and electron−phonon coupling strengths have been observed recently in other lightly doped oxides including TiO_2_, Sr_2−*x*_La_*x*_TiO_4_, as well as ZnO- and SrTiO_3_-based two-dimensional electron gases^4–6,8,27–29^. Their observation here, within the markedly different system of the bulk-doped three-dimensional and spin-polarised electron pocket of EuO, suggests that polaron formation is likely universal to lightly doped polar oxides.

### Doping-dependent spectral function

**Fig. 3:**
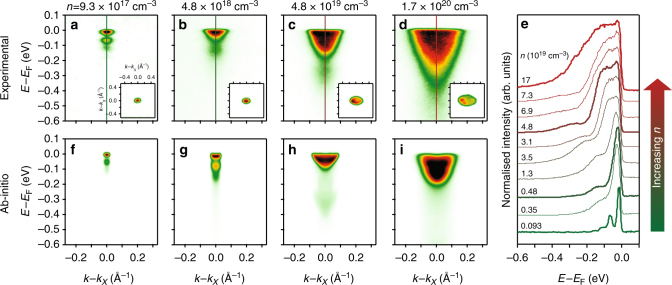
Doping-dependent plasmonic polarons. **a**–**d** Evolution of the measured spectral function of Eu_1−*x*_Gd_*x*_O with increasing charge carrier doping, showing not only a strong increase in band filling of the quasiparticle band, but also a substantial evolution of the satellite peak structure. The insets show Fermi surface contours (*hν* = 137 eV), indicating the increasing doping. While replica bands can still be observed to high doping, as clearly evident as peak-dip-hump structures in measured EDCs (**e**), these show a strong broadening and blue-shift relative to the quasiparticle peak with increasing doping. **f**–**i** Our ab initio calculations reproduce this general trend when both electron−phonon and electron−plasmon interactions are considered, identifying the hump feature in the higher-density samples as arising from plasmonic polarons

**Fig. 4:**
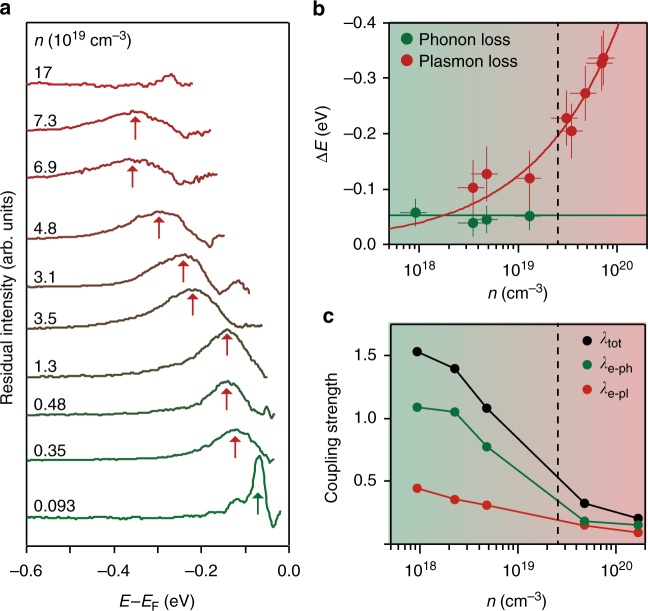
Tuning and disentangling the interplay of electron−phonon and electron−plasmon coupling. **a** Normalised residual intensity plot of EDCs at the centre of the electron pocket (see Supplementary Fig. 3), revealing a clear satellite structure that shifts to higher binding energy with increasing charge carrier doping. **b** Fits to the measured raw EDCs reveal that the separation of this satellite from the quasiparticle band follows the functional form of a plasmon mode (see Methods), while an additional weak satellite feature is found to remain at a constant energy for the lower-doped samples, which we attribute to a phonon-induced replica band. Error bars reflect the uncertainty in extracting the Luttinger area and satellite binding energies from the experimental measurements, and incorporate statistical errors in peak fitting as well as systematic experimental uncertainties. **c** Decomposition of the coupling strength to phonon and plasmon modes from the ab initio calculations reveals a rich carrier-density-driven crossover in the underlying nature of dominant many-body interactions in this system

We show in Fig. 3 how the spectral function evolves with increasing carrier doping. By increasing the density of the Gd^3+^ dopants, the band filling can be controllably increased, as evidenced by the increased quasiparticle bandwidth as well as the larger Fermi surface volume, shown inset in Fig. 3a–d. With even a small increase in carrier density, however, the pronounced multi-peak satellite structure observed in the lowest-doped sample is no longer apparent. This points to a rapid reduction in the electron−phonon coupling strength, a phenomenon which we return to below. Nonetheless, a broadened satellite peak is still observed below the quasiparticle band (Fig. 3b, c), evident as a hump in EDCs (Fig. 3e) which persists over at least two orders of magnitude increase in carrier density. To demonstrate this more clearly, we show in Fig. 4a the residual of the measured EDC intensity after subtraction of a background function accounting for the quasiparticle peak intensity (see also Supplementary Fig. 3).

The satellite peak broadens with increasing charge carrier doping, but remains clearly resolved up to a carrier density *n* ≈ 10^20^ cm^−3^. At the same time, the satellite exhibits a pronounced shift to higher binding energy with increasing doping. The shift is much faster than the increase in filling of the conduction band. Indeed, from fits to the measured data, we find that the separation of this hump feature from the band bottom of the quasiparticle band grows with a $\sqrt n$ dependence, where *n* is the three-dimensional electron density (Fig. 4b). This indicates electron−boson coupling to a mode which hardens with increasing carrier density. This is in striking contrast to the expectations for a phonon mode, which should be nearly carrier density independent. Instead, it agrees well with the functional form of the mode energy expected for a plasmon (red line in Fig. 4b).

The satellite features we observe here for our higher-density samples therefore point to the formation of plasmonic polarons, where the conduction electrons become dressed by charge-density fluctuations of their own electron gas^30^. This interpretation is confirmed by our ab initio calculations, where we are able to treat electron−phonon and electron−plasmon coupling on an equal footing. Our obtained spectral functions (Fig. 3f–i) reproduce the general trends observed experimentally, also yielding plasmonic polaron satellites shifted below the quasiparticle band by the conduction electron plasmon energy. Given that EuO is a half-metal for the levels of doping investigated here, these plasmon−polarons must necessarily also be spin-polarised. Indeed, the spin-polarised conduction band of EuO has led to significant interest in using this material for spin-injection in spintronics applications^17^. The polaronic nature of the spin-polarised charge carriers in EuO, and consequent limited intrinsic carrier mobilities that would be expected, should be carefully considered for such applications. More generally, the excellent agreement that we find between our experimental and ab initio spectral functions for a real, complex, multi-orbital and magnetic system such as EuO suggests opportunities to exploit such advanced calculation schemes for not only understanding, but increasingly predicting, the interacting electronic states and properties of functional materials.

### Tuneable plasmon polarons

We focus below on the origin, and unique properties, of the plasmon−polarons discovered here. For a three-dimensional electron gas, the plasmon dispersion, *ω*(**q**), remains gapped in the long-wavelength limit (*ω*(|**q**|→0) = *ω*_p_, where *ω*_p_ is the plasma frequency), while the electron−plasmon coupling strength goes as 1/|**q**|. Our direct observation of satellite structures spaced by the plasma energy here indicates that this is sufficient to generate well-defined replica bands, similar to those generated by the Fröhlich electron−phonon interaction. We note that this is different to the occurrence of sharp plasmon-mediated features in the spectral function of graphene, which rely upon pseudospin conservation and phase-space restrictions from matching the group velocity of plasmon and band dispersions^31^ which would not be expected in the current system. Instead, the plasmon–polarons observed here can be expected as a generic feature in the low- to medium-doping limit of a doped three-dimensional semiconductor. Moreover, we note that the polarons observed here have markedly different characteristics to plasmonic polaron band structures that have been predicted to occur via excitation of high-energy valence plasmons^30,32,33^, with experimental signatures recently observed in silicon^33^ and graphite^34^. In such systems, the plasmon energy scales are ~3 orders of magnitude larger than the other excitations in the system. In contrast, the plasmons considered here have comparable energy to the Fermi energy, and so can be expected to have a much more dramatic influence on the low-energy properties of the system, such as enhancing the quasiparticle mass and limiting charge carrier mobilities.

Moreover, the conduction-electron plasmons here are highly tuneable via charge carrier doping, with a characteristic mode energy that can be driven into resonance with, for example, phonon modes of the system as shown in Fig. 4. When close in energy, the two bosonic modes will in general couple to each other, leading to hybrid phonon−plasmon polaritons. Such a mode coupling is not explicitly considered in our calculations, although would be consistent with our experimentally determined satellite structure (Supplementary Fig. 4). Our study thus motivates the development of theoretical approaches and targeted experiments to investigate the polaronic signatures that might be expected in this intriguing regime.

Even without considering such mode hybridisation, our ab initio calculations shown in Fig. 4c already reveal a rich doping-dependent interplay of the coupling strength of charge carriers to different bosonic modes in the system. For the lowest carrier density investigated, a large electron−phonon coupling of *λ*_e−ph_ > 1 is obtained, which is the dominant coupling in the system. Given this, and that the plasma frequency is very small for this level of charge carrier doping, the multi-peak satellite structure observed experimentally thus predominantly arises due to electron−phonon interactions (cf. Figs. 2b and 3f). In this regime (plasma energy much smaller than the phonon mode energy), the system hosts Fröhlich polarons^35^.

With increasing carrier density, the plasma frequency becomes larger than the phonon frequency (Fig. 4b). The electron−phonon interaction therefore becomes efficiently screened^23^, and so the electron−phonon coupling strength shows a rapid drop-off with increasing charge carrier doping. Nonetheless, a satellite peak remains visible in both our calculations (Fig. 3g) and in fits to our measured experimental data (Fig. 4b) until the Fermi energy becomes comparable to the phonon mode frequency (approximately at the dashed line in Fig. 4b). Beyond this point, the system moves into a Fermi liquid regime^23^, and the electron−phonon interaction instead leads to a more conventional kink in the band dispersion near to the Fermi level. Weak signatures of this are visible in our experimental data (Fig. 3c, d), although they are somewhat obscured by broadening due to a poor *k*_*z*_ resolution resulting from the inherent surface sensitivity of photoemission.

**Fig. 5:**
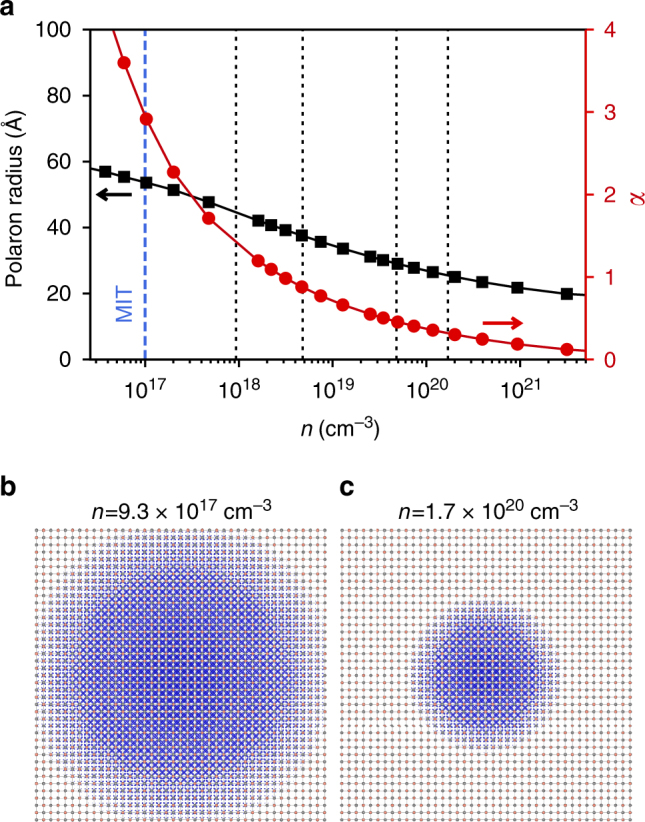
Plasmonic polaron structure. **a** Evolution of the electron−plasmon coupling constant *α* (red symbols) and of the plasmonic polaron radius (black symbols) with carrier density. The black-dashed lines indicate the experimental doping levels from Fig. 3, while the blue line marks the critical density at the metal-insulator transition. **b**, **c** Square modulus of the polaron wavefunction for *n* = 9.3×10^17^ cm^−3^ (**b**) and *n* = 1.7×10^20^ cm^−3^ (**c**). For clarity the wavefunctions are represented only in the *xz* plane, with 20 replicas of the unit cell shown along each direction

Despite these qualitative changes in the nature of the electron−phonon interactions, the electron−plasmon coupling strength evolves more smoothly with increasing charge carrier density (Fig. 4c). It thus plays a more dominant role at somewhat higher carrier densities, where the electron−phonon interaction is more efficiently screened. It still, however, displays a pronounced dependence on charge carrier doping in the system. To investigate this over a wider carrier density range, we show in Fig. 5a the effective electron−plasmon coupling constant, *α*, and the plasmonic polaron radius derived from the self-energy for a homogeneous electron gas with the same effective mass and dielectric permittivity as EuO (see Methods). The increase of *α* with decreasing carrier density suggests that a strong coupling regime between electrons and plasmons may be approached at low doping concentrations. In practice, the critical doping density which marks the onset of a Mott metal−insulator transition, poses a strict limit to the highest coupling that will be achievable in practice, since below this value the system becomes insulating and plasmons cannot be excited. The critical density in EuO, for example, is ~10^17^ cm^−3^ and it is marked by the vertical dashed line in Fig. 5a. At this doping, we find *α* ≃ 2.9 and a polaron radius of 53 Å. These values and their dependence on carrier concentrations are compatible with the results obtained from our first-principles calculations at the experimental doping densities (marked by dotted lines in Fig. 5a) reported in Supplementary Table 1.

Interestingly, we show in Fig. 5a that the polaron radius decreases with increasing carrier density. In fact, over the doping range considered in our experiments, the plasmonic polaron radius approximately doubles, as illustrated in Fig. 5b, c. This is in striking contrast to the expected behaviour known from phononic polarons, where the polaron radius decreases with increasing coupling strength (i.e., increases with increasing carrier density)^36^. This unconventional behaviour results from the strong dependence of the plasmon energy on carrier concentration, and may lead to unconventional trends in doping-dependent mobilities. Moreover, this further points to the highly tuneable nature of plasmonic polaron states, whereby a broad spectrum of electron−boson coupling regimes can be explored by tuning the carrier concentrations.

## Discussion

We stress that our findings should not be specific to EuO, but rather a general feature of band insulators where conductivity can be induced by dilute charge carrier doping. They suggest substantial opportunity to engineer the relative importance of different bosonic modes, and may allow triggering or controlling instabilities of the collective system via electron−plasmon as well as electron−phonon interactions. Indeed, superconductivity in SrTiO_3_/LaAlO_3_ interface 2D electron gases has recently been argued to emerge from a phonon polaron liquid, with its superconducting dome linked to a doping-dependent strength of electron−phonon coupling^5^. A similar superconducting instability could generically be expected to occur for the plasmon−polarons introduced here.

As shown above, the coupling strength for both phonon and plasmon polarons decreases with increasing doping. In the phononic case, the mode energy is fixed, and so this decrease in coupling strength must lead to a decrease in superconducting transition temperature as the doping is increased. In contrast, for the plasmonic case, while the coupling strength still decreases with increasing carrier doping, the influence on *T*_c_ should be partially offset by a hardening of the relevant mode energy. This could even lead to a doping-dependent crossover from phonon- to plasmon-mediated superconductivity with increasing charge carrier doping. While such considerations would not be relevant for EuO as studied here, due to its ferromagnetic nature, our results point to the intriguing possibility to stabilise unusual doping-dependent superconducting instabilities in, for example, lightly doped oxide semiconductors. Furthermore, they highlight the complexity of charge-carrier doping in oxides even in the absence of strong electronic correlations, opening routes to the targeted design of their materials properties.

## Methods

### Molecular-beam epitaxy

The EuO thin films were grown by MBE utilising a Createc miniMBE system^37^ installed on the I05 beamline at Diamond Light Source, UK. The films were grown on YAlO_3_ substrates in an absorption-controlled, or distillation^38^, growth mode at a temperature of 425 °C, using an Eu partial pressure of *p*_Eu_ ≈ 2.3 × 10^−7^ mbar and a molecular oxygen partial pressure of $p_{{\mathrm{O}}_{\mathrm{2}}} \approx 2.0 \times 10^{ - 8}\,{\mathrm {mbar}}$, as measured by a beam flux monitor. Gd dopants were introduced by exposing the films to a Gd partial pressure of *p*_Gd_ ≈ 6.3 × 10^−9^ mbar during growth, and shuttering the Gd source (for four equal length periods within each monolayer of EuO growth) to further reduce the incorporated Gd concentration. The films were monitored in situ using reflection high energy electron diffraction (see Supplementary Fig. 5), from which the inverse growth rate was determined to be ≈120 s per monolayer. The total thickness of the grown films is ≈20 nm, thick enough to ensure that their electronic structure is bulk-like^39^. Following growth, the films were transferred under ultra-high vacuum to the HR-ARPES end-station (see below). After the ARPES measurements, they were further characterised by low-energy electron diffraction (see Supplementary Fig. 5) and x-ray photoelectron spectroscopy (Supplementary Fig. 6), which indicated their high crystalline and chemical quality. We note that our undoped samples are highly insulating, pointing to negligible oxygen vacancy concentrations, while our x-ray photoelectron spectroscopy (XPS) measurements indicate an Eu^2^+ charge state, indicative of the growth of stoichiometric EuO. The samples were then capped with 5–15 nm of amorphous silicon (*p*_Si_ ≈ 2.5×10^−8^ mbar), allowing these air-sensitive samples to be removed from the ultra-high vacuum environment. A subset of the films were then further probed by superconducting quantum interference device magnetometry to probe their magnetic properties and x-ray absorption spectroscopy to assess the Gd doping (Supplementary Fig. 7). These measurements confirmed material and magnetic properties of our grown EuO films that are in good agreement with previous studies of this compound. We also show in Supplementary Fig. 8 temperature-dependent ARPES measurements of a moderate carrier density sample. The majority band (occupied at low temperature) can be seen moving up through the Fermi level upon increasing through the Curie temperature, driving a temperature-dependent metal−insulator transition as a result of the loss of exchange splitting. This is fully consistent with the expected presence of spin-polarised exchange-split bands at low temperature, entirely in line with our spin-polarised DFT calculations (Fig. 1b).

### Angle-resolved photoemission

In situ ARPES was performed using the High-Resolution ARPES instrument (HR-ARPES) of Diamond Light Source, UK. Measurements were performed at temperatures of ≈20 K or below using p-polarised synchrotron light. A Scienta R4000 hemispherical electron analyser was used, with a vertical entrance slit and the light incident in the horizontal plane. Photon energies of 48 and 137 eV was used. For an inner potential of 15 eV, these correspond to measured dispersions which cut centrally through the conduction band Fermi surface of EuO along *k*_*z*_, with an in-plane dispersion along the short axis of the elliptical Fermi pocket as shown in Supplementary Fig. 1. To determine the carrier density of the doped films, we extracted the Luttinger volume of their measured Fermi surfaces. This is complicated by the three-dimensional nature of these Fermi pockets, and the inherently poor *k*_*z*_ resolution in ARPES arising from its surface sensitivity. We therefore simulated the measured Fermi surface including the effects of *k*_*z*_ broadening, and compared this to our experimental data to determine the correct carrier density (Supplementary Fig. 9). This carrier density enters into the fit for the plasma frequency of a three-dimensional electron gas, $\omega _p = \sqrt {ne^2{\mathrm{/}}\varepsilon _0\varepsilon _\infty m^ \ast },$ where *ε*_0_ is the dielectric permittivity of free space, and *ε*_∞_ is the dielectric constant of EuO (4.5 ^24^). m^*^ is the effective mass, which is treated as a fit parameter in our analysis (Fig. 4b), from which we find a value of m^*^ = 0.2 ± 0.1 *m*_e_ within the range of previous estimates of the effective mass of EuO^40–42^.

### First-principles calculations

Density-functional theory calculations including Hubbard corrections (DFT + *U*)^43^ for the low-temperature ferromagnetic phase of EuO were performed using Quantum ESPRESSO^44^. We employed the Perdew, Burke and Ernzerhof (PBE)^45^ exchange-correlation functional, an effective onsite Coulomb parameter *U*_*f*_ = 6 eV for the Eu 4*f* states, and *U*_*p*_ = 3 eV for the O 2*p* states. We used norm-conserving pseudopotentials, a plane wave kinetic energy cutoff of 150 Ry, and a 8 × 8 × 8 uniform *k*-point mesh to sample the Brillouin zone. Maximally localised Wannier functions were constructed starting from a 4 × 4 × 4 uniform **k** grid. The effect of electron doping was included in the rigid-band approximation. The lattice vibrational properties were calculated using the projector augmented wave (PAW) method^46^, and effective Coulomb parameters *U*_*f*_ = 8.3 eV and *U*_*p*_ = 4.6 eV which yield the same band gap calculated with norm-conserving pseudopotentials. Convergence was ensured by using a kinetic energy cutoff of 70 Ry. The phonon dispersions were obtained by finite differences in a 6 × 6 × 6 supercell, using atomic displacements of 0.01 Å. The longitudinal optical-transverse optical (LO-TO) splitting was accounted for as in ref. ^47^, using the calculated Born effective charges from Ref. ^48^.

The first-principles spectral functions were obtained from the cumulant expansion method^23,49,50^ using the electron−phonon and electron−plasmon self-energy as implemented in the EPW code^51–53^ as a seed:

1 $$\Sigma _{{n} {\bf{k}} } (\omega )\,=\, \mathop {\sum}\limits_{mv} {{\int} {\frac{{{\mathrm d}{\bf{q}}}}{{\Omega _{{\mathrm{BZ}}}}}\left| {g_{mn\nu }({\bf{k}},{\bf{q}})} \right|^2} } \\ \times \left[ {\frac{{n_{{\bf{q}}\nu } + f_{m{\bf{k}} + {\bf{q}}}}}{{\hbar \omega - \varepsilon _{m{\bf{k}} + {\bf{q}}} + \hbar \omega _{{\bf{q}}\nu } - i\eta }}\,+\,\frac{{n_{{\bf{q}}\nu } + 1 - f_{m{\bf{k}} + {\bf{q}}}}}{{\hbar \omega - \varepsilon _{m{\bf{k}} + {\bf{q}}} - \hbar \omega _{{\bf{q}}\nu } - i\eta }}} \right].$$

Here, *η* is a positive infinitesimal, *f*_*m***k** + **q**_ and *n*_**q***ν*_ are Fermi−Dirac and Bose−Einstein occupations, respectively, *ε*_*m***k** + **q**_ is the electron energy, and $\hbar \omega _{{\bf{q}}\nu }$ is the energy of a plasmon/phonon with wavevector **q**. The coupling matrix elements due to electron−plasmon and electron−phonon coupling were computed as in ref. ^52^ and ref. ^54^, respectively. For the electron−phonon coupling, dynamical screening arising from the added carriers in the conduction band was taken into account by using nonadiabatic matrix elements^23^: $g_{mnv}^{{\mathrm{NA}}}\left( {{\bf{k}}{\mathrm{,}}{\bf{q}}} \right)\,=\,g_{mnv}\left( {{\bf{k}},{\bf{q}}} \right){\mathrm{/}} \varepsilon \left( {{\bf{q}}{\mathrm{,}}\omega _{{\bf{q}}v} + i{\mathrm{/}}\tau _{n{\bf{k}}}} \right)$. Here *ε*(**q**,*ω*) is the Lindhard dielectric function for a spin-polarised homogeneous electron gas with effective mass m^*^ and dielectric permittivity *ε*_∞_ of EuO, and $\hbar /\tau _{n{\bf{k}}}$ is the electron lifetime near the band edge, taken to be 50 meV. Finite resolution effects were accounted for by applying two Gaussian masks of widths 20 meV and 0.015 Å^−1^, and by integrating the spectral function along the out-of-plane direction *k*_*z*_. The temperature broadening at the Fermi level was included via a Fermi–Dirac distribution at *T* = 20 K. The electron−plasmon and electron−phonon coupling strengths *λ* were extracted from the self-energy via $\lambda\,=\,- \hbar ^{ - 1}\left. {\partial {\mathrm{R}}{\mathrm e}{\kern 1pt} \Sigma ({\bf{k}}_{\mathrm{F}},\omega ){\mathrm{/}}\partial \omega } \right|_{\varepsilon _{\mathrm{F}}}$^19^. The effective electron−plasmon coupling constants *α* were obtained from the mass renormalisation 1 + *λ*_e−pl_^22^, whereas the plasmonic polaron radius was estimated following ref. ^55^: $r_p\,\simeq\,\left( {3{\mathrm{/}}0.44\alpha } \right)^{\frac{1}{2}}(2m\omega _{{\mathrm{pl}}}{\mathrm{/}}\hbar )^{ - \frac{1}{2}}$. The polaron wavefunction was calculated as the product of the lattice-periodic component of the Kohn−Sham eigenstate at the conduction-band bottom and a Gaussian with isotropic width *σ* corresponding to the polaron radius.

### Code availability

The calculations were performed using the open-source software projects Quantum ESPRESSO, EPW, and Wannier90, which can be downloaded free of charge from www.quantum-espresso.org, epw.org.uk, and www.wannier.org, respectively. Input files and calculation workflows can be downloaded from the GitHub repository https://github.com/mmdg-oxford/papers.

### Data availability

The data that underpins the findings of this study are available at 10.17630/4e82a731-57c6-4cf5-b8c2-841486b8dbde.

## Electronic supplementary material

Supplementary Information

Peer Review File
