# Supplementary material for: Crossover from lattice to plasmonic polarons of a spin-polarised electron gas in ferromagnetic EuO
Source: Nat Commun. 2018 Jun 13;9:2305. doi: 10.1038/s41467-018-04749-w (PMC5998015; doi:10.1038/s41467-018-04749-w)
Supplement: Supplementary file 1 — Supplementary Information [file 41467_2018_4749_MOESM1_ESM.pdf]

# Supplementary Information: Crossover from Lattice to Plasmonic Polarons of a Spin-Polarised Electron Gas in Ferromagnetic EuO

J. M. Riley,[1,2] F. Caruso,[3,4] C. Verdi,[3] L. B. Duffy,[5,6]
M. D. Watson,[1,2] L. Bawden,[1] K. Volckaert,[1] G. van der Laan,[2]
T. Hesjedal,[5] M. Hoesch,[2,*] F. Giustino,[3,*] and P. D. C. King[1,*]

[1]*SUPA, School of Physics and Astronomy, University of St. Andrews, St. Andrews KY16 9SS, United Kingdom*
[2]*Diamond Light Source, Harwell Campus, Didcot, OX11 0DE, United Kingdom*
[3]*Department of Materials, University of Oxford, Parks Road, Oxford, OX1 3PH, United Kingdom*
[4]*Institut für Physik and IRIS Adlershof, Humboldt-Universität zu Berlin, Berlin, Germany*
[5]*Department of Physics, University of Oxford, Oxford, OX1 3PU, United Kingdom*
[6]*ISIS, STFC, Rutherford Appleton Laboratory, Didcot, OX11 0QX, England, United Kingdom*

(Dated: May 22, 2018)

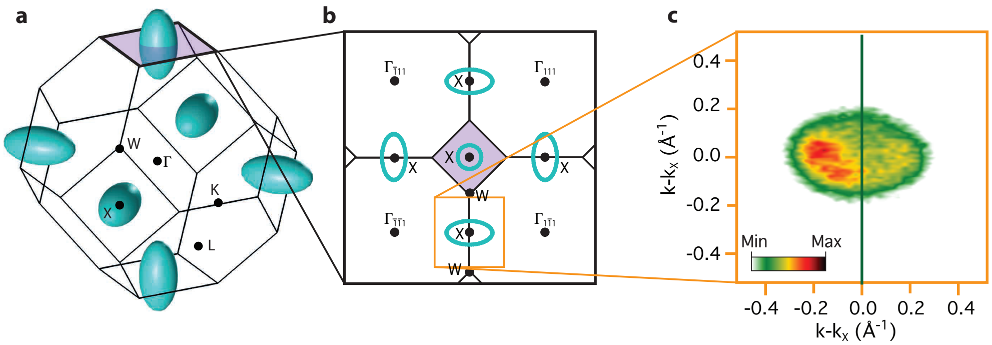


Supplementary Figure 1. **Fermi Surface of electron-doped EuO** (a) DFT calculated three-dimensional Fermi surface obtained from a rigid shift of the Fermi level into the conduction band of EuO. Elliptical electron pockets are found centred at each X-point of the three-dimensional Brillouin zone. (b) For a plane centered at the Brillouin zone face along the out-of-plane momentum direction ($k_z$=$2\pi/a$), these form circular and elliptical pockets within the square and octagonal cuts of the first and neighbouring Brillouin zones, respectively. (c) A measured ARPES Fermi surface in this plane, over the orange region shown in (b). The red line depicts the direction used for the measured ARPES cuts presented in the main text.

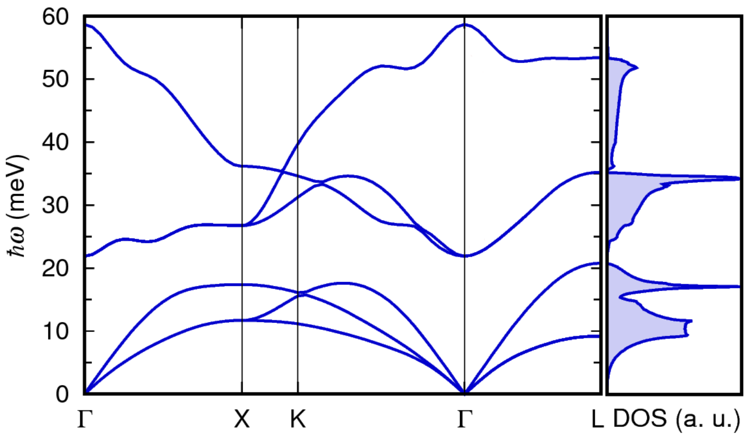


Supplementary Figure 2. **Phonon dispersion of EuO.** Calculated phonon dispersions of EuO, with the phonon density of states shown on the right. The Born effective charges used to compute the LO-TO splitting are taken from the calculations in Ref.[1]: $Z^* = 2.65$ for the Eu atoms and $Z^* = -2.65$ for the O atoms.

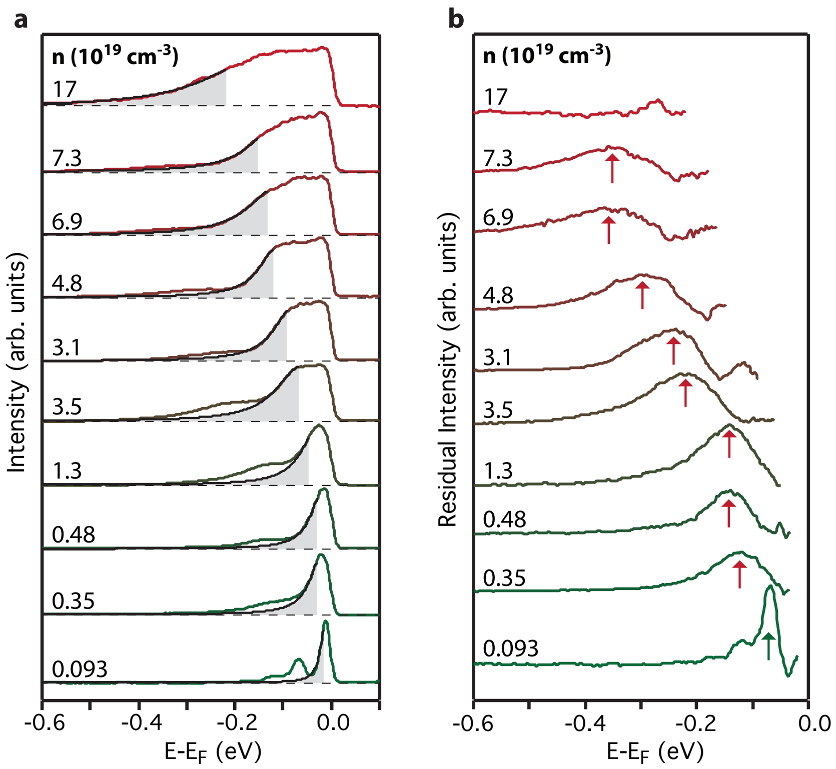


Supplementary Figure 3. **Visualisation of the plasmon loss satellites.** (a) Energy distribution curves (EDCs) through the centre of the conduction bands (blue to red coloured lines) as a function of doping, as shown in Fig. 3(e) of the main text. To extract the intensity contributed from the plasmon loss satellite, a Lorentz function (black line with grey shading underneath) is fit to the high binding energy tail and the rising edge of the spectral weight towards shallower binding energy. This serves as a background function for the plasmon loss satellite. The residual intensity of the measured EDCs after subtraction of this background intensity is shown in (b), and reproduced in Fig. 4(a) of the main text.

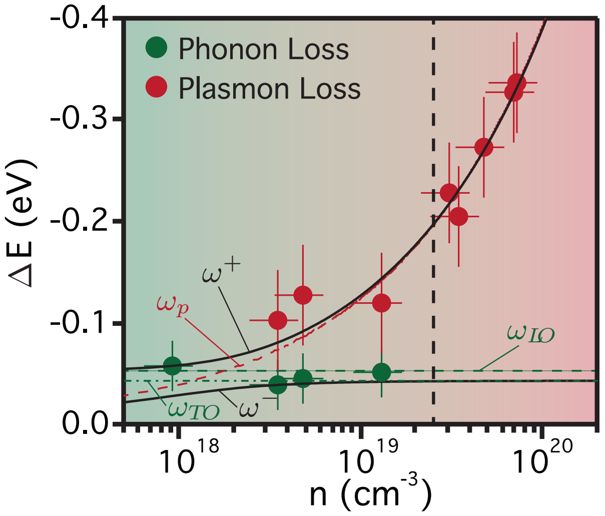


Supplementary Figure 4. **Polariton Branches.** Quasiparticle-satellite peak separation extracted experimentally, reproduced from Fig. 4(b) of the main text. Error bars reflect the uncertainty in extracting the Luttinger area and satellite binding energies from the experimental measurements, and incorporate statistical errors in peak fitting as well as systematic experimental uncertainties. Dashed lines show the expected density-dependent relations for a pure plasmon mode ($\omega_p$) as well as longitudinal ($\omega_{LO}$) and transverse ($\omega_{TO}$) optical phonon modes taken from Ref. [2]. Solid lines show the expected behaviour when allowing a hybridisation of these modes forming polariton branches[3]: $\omega_{\pm}^2 = \frac{1}{2}(\omega_p^2 + \omega_{LO}^2) \pm \frac{1}{2}\sqrt{(\omega_p^2 + \omega_{LO}^2)^2 - 4\omega_p^2\omega_{TO}^2}$.

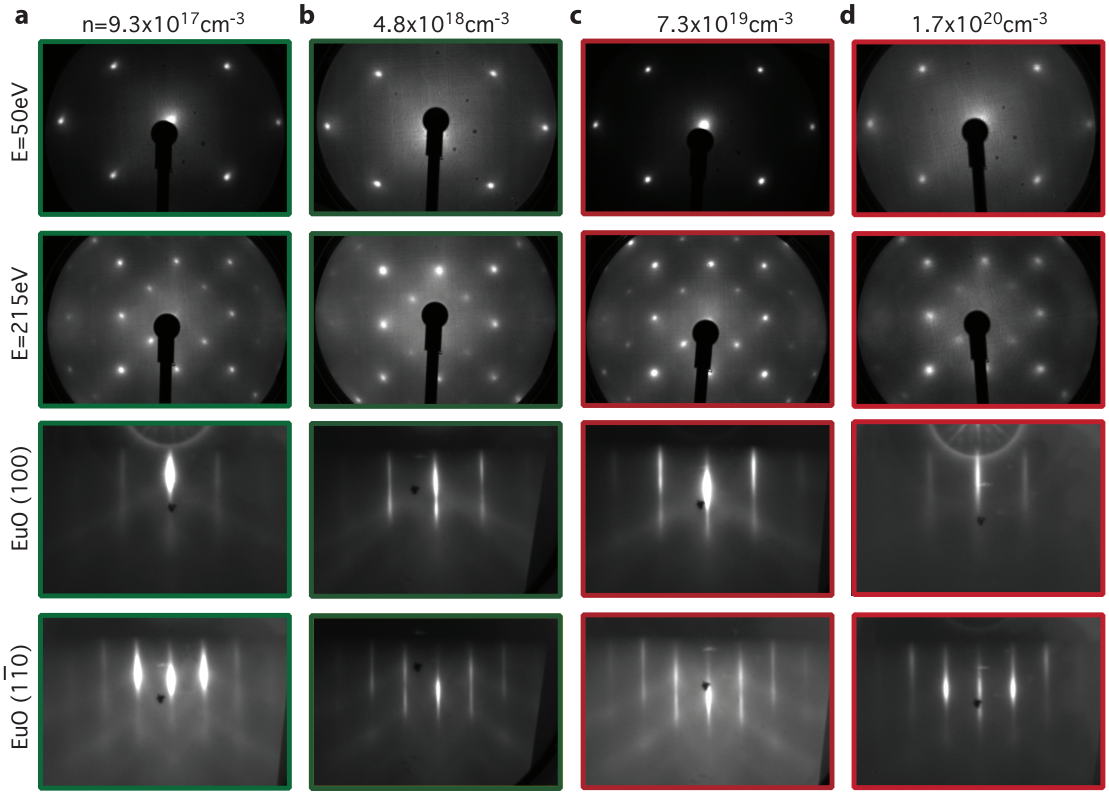


Supplementary Figure 5. **Surface structure of EuO thin films.** (a) Low-energy electron diffraction (LEED, top two rows) images of lightly doped ($n = 9.3 \times 10^{17}$ cm$^{-3}$) EuO, showing sharp diffraction spots, and a symmetry consistent with its rocksalt crystal structure. An incident electron energy of 50 eV (top panel) and 215 eV (second panel) was used. Reflection high-energy electron diffraction (RHEED, bottom two panels) patterns along the EuO (100) and (1$\bar{1}$0) azimuths, respectively, measured in the MBE chamber immediately after growth. (b-d) Same as in (a) but with increasing carrier density ($4.8 \times 10^{18}$, $7.3 \times 10^{19}$, and $1.7 \times 10^{20}$ cm$^{-3}$, respectively), showing how the high crystalline quality is maintained across the full range of doping/carrier concentrations considered here.

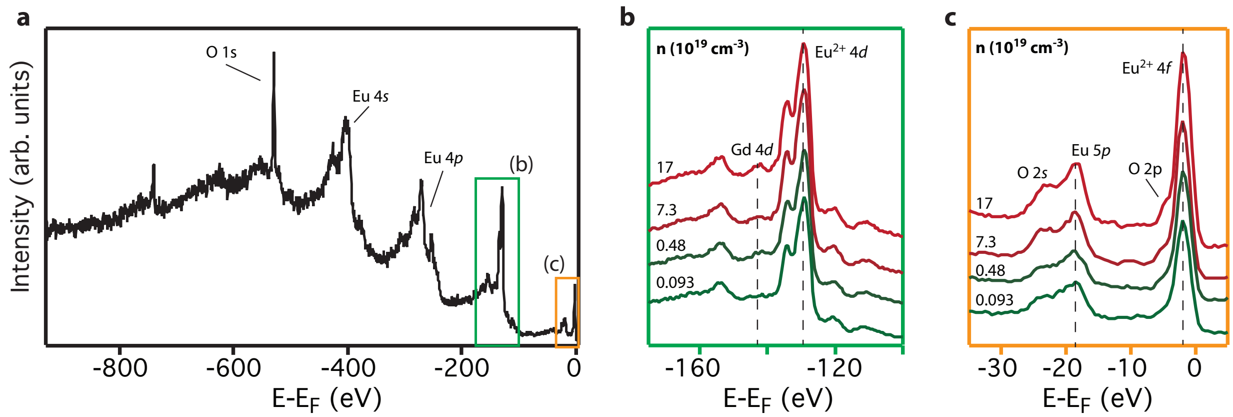


Supplementary Figure 6. **Chemical composition of EuO thin films.** (a) X-ray photoemission spectroscopy (XPS) spectra of Gd-doped EuO thin films, performed *in situ* following completion of the ARPES measurements. (b) Higher resolution measurements of the Eu 4$d$ core levels indicate that the Eu is in a 2+ valence state consistent with growth of europium monoxide, with no signs of the presence of $Eu^{3+}$ which would be characteristic of over-oxidised samples. The Gd 4$d$ core level increases in intensity with increasing nominal Gd content from the growth. (c) The presence of $Eu^{2+}$ is further supported from the shallow Eu 4$f$ and 5$p$ as well as O 2$p$ and 2$s$ core levels/valence bands.

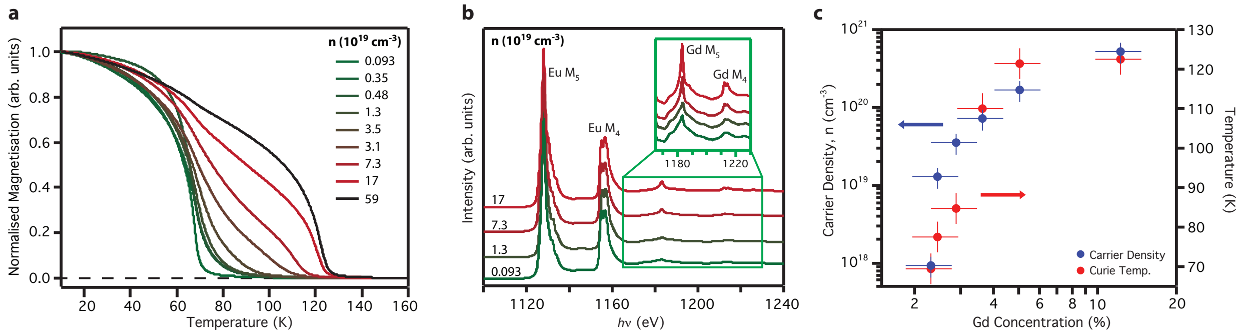


Supplementary Figure 7. **Doping-dependent magnetic properties.** (a) Temperature-dependent magnetisation obtained from SQUID measurements, revealing a large increase in Curie temperature ($T_c$) with increased Gd concentration. The Curie temperature is seen to approximately saturate around $T_c = 123$ K at the highest carrier densities, consistent with previous studies.[4] (b) X-ray absorption spectroscopy (XAS) measurements of the Eu and Gd $M_{4,5}$ absorption edges, used to calculate the Gd concentration. (c) Comparison of Gd concentration to carrier density and Curie temperature. The Gd dopants are not all active, with an activation of $\sim 15\%$ at the highest doping levels, again consistent with previous studies.[4] Error bars reflect the uncertainty in extracting the Luttinger area from the ARPES and Gd:Eu spectral weight from the XAS, and incorporate statistical errors in peak fitting as well as systematic experimental uncertainties.

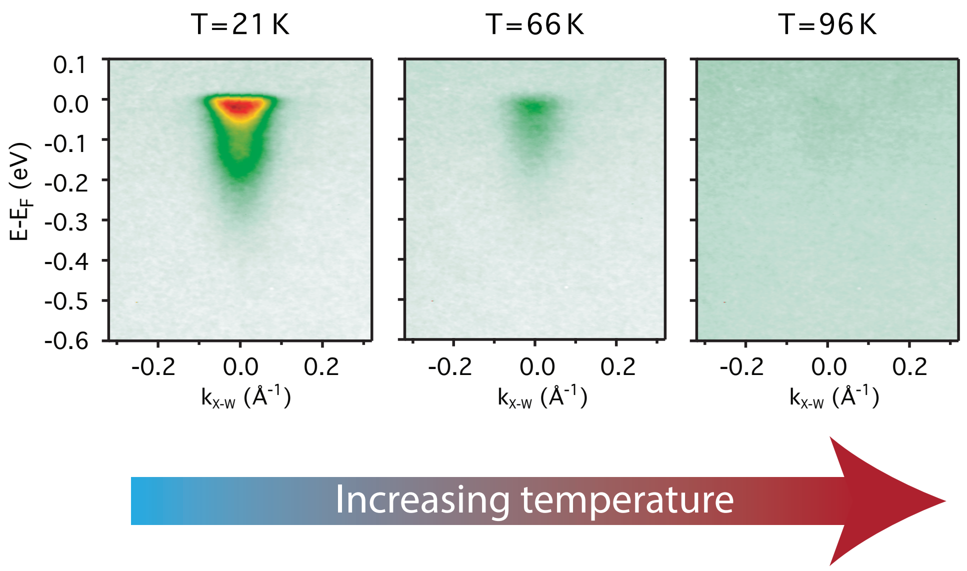


Supplementary Figure 8. **Temperature-dependent electronic structure.** Temperature-dependent spectral function measured from a sample with carrier density $n = 1.3 \times 10^{19}$ cm$^{-3}$. The upwards shift and complete depopulation of the majority band is evident upon moving through the Curie temperature (78 K), consistent with a fully spin-polarised conduction band at low temperature.

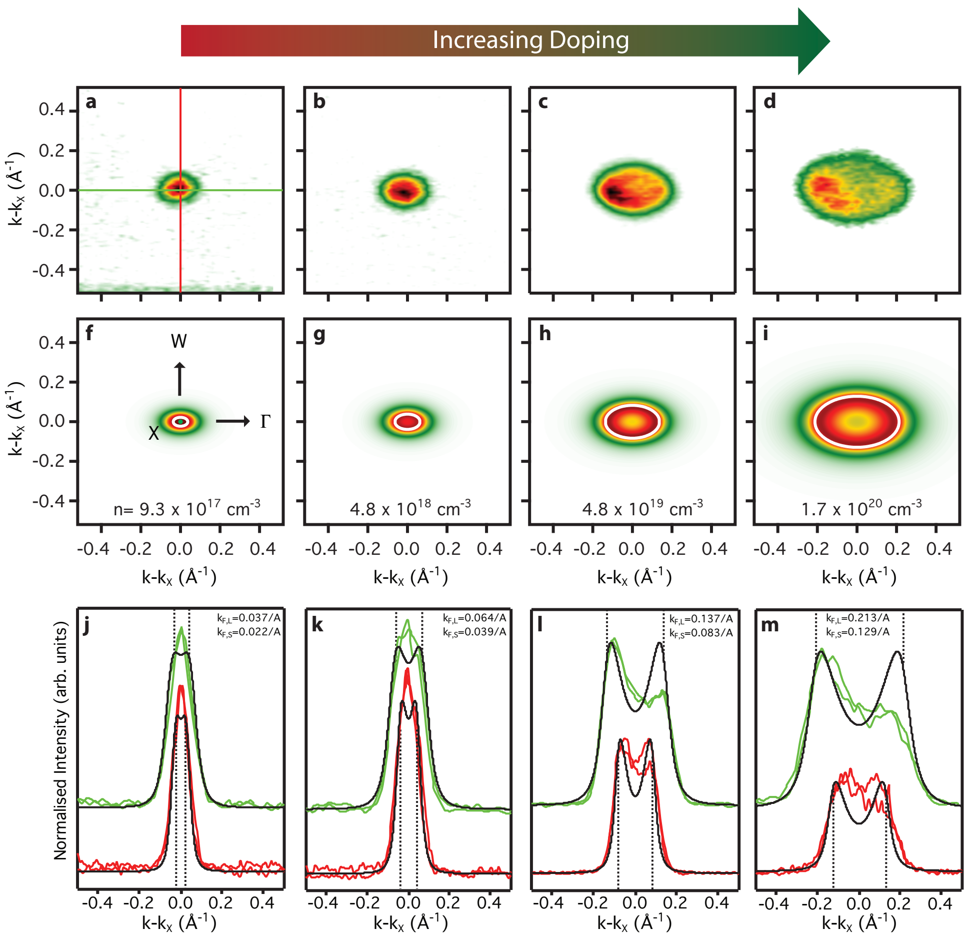


Supplementary Figure 9. **Determination of carrier density from measured Fermi surface contours.** (a) ARPES ($h\nu$=137 eV) Fermi surface contour of the electron pocket centred at X. (b-d) Same as (a) but with increasing carrier density revealing the increase in size of the diffuse spectral weight with electron doping. (f-i) Simulation of the spectral intensity for the carrier densities shown in the panels above. The measured spectral intensity is calculated as $I(k_x, k_y) \propto \left(\sum_{k_z} \frac{I(k_z)}{E_0(k_x,k_y,k_z)^2+\epsilon}\right) \otimes G(k_x, k_y)$ where $E_0$ is the bare band dispersion calculated assuming a parabolic effective mass model, $\epsilon$ is a constant imaginary part of the self-energy and $G(k_x, k_y)$ is a 2D Gaussian function to account for experimental broadening. The sum over $k_z$ accounts for the poor $k_z$ resolution inherent to ARPES where $I(k_z)$ is a Gaussian $I(k_z) = \frac{1}{2\pi\lambda} \frac{1}{(k-k_{z,0})^2+(1/2\lambda)^2}$ representing the integration over $k_z$ in our measured spectra, and $\lambda$ is the inelastic mean free path determined by the TPP-2M algorithm.[5] The bare band contours are overlaid in white. (j-m) Comparison of momentum distribution curves (MDCs) at the Fermi level between the simulations (black) and measured Fermi surface contours aligned along the X-W (red) and X-Γ (green) directions. Dashed lines denote the Fermi wavevector along both directions.

Supplementary Table I. Electron-plasmon coupling constant $\alpha$ and plasmonic polaron radius $r_{\mathrm{p}}$ calculated for the four doping concentrations of Fig. 3(a)-(d) of the main text. The values of $\lambda_{\mathrm{e\text{-}pl}}$ reported correspond to the ones in Fig. 4(c) in the main text.

| $n$ (cm$^{-3}$) | $\lambda_{\mathrm{e\text{-}pl}}$ | $\alpha$ | $r_{\mathrm{p}}$ (Å) |
|---|---|---|---|
| $9.3 \times 10^{17}$ | 0.44 | 1.8 | 36 |
| $4.8 \times 10^{18}$ | 0.31 | 1.4 | 27 |
| $4.8 \times 10^{19}$ | 0.15 | 0.8 | 21 |
| $1.7 \times 10^{20}$ | 0.09 | 0.5 | 18 |

## Supplementary References

* To whom correspondence should be addressed: moritz.hoesch@gmail.com, feliciano.giustino@materials.ox.ac.uk & philip.king@st-andrews.ac.uk

[1] R. Pradip *et al.*, Lattice Dynamics of EuO: Evidence for Giant Spin-Phonon Coupling *Phys. Rev. Lett.* **116**, 185501 (2016).

[2] J.D. Axe, Infrared dielectric dispersion in divalent europium chalcogenides. *J. Phys. Chem. Solids* **30**, 1403 (1969).

[3] G.D. Mahan, Many-Particle Physics. 3rd ed. (Kluwer Academic, Dordrecht, The Netherlands, 2000).

[4] T. Mairoser *et al.*, Is There an Intrinsic Limit to the Charge-Carrier-Induced Increase of the Curie Temperature of EuO? *Phys. Rev. Lett.* **105**, 257206 (2010).

[5] Tanuma, S. Powell, C. J. and Penn, D. R. Calculations of electron inelastic mean free paths. *Surf. Interface Anal.* **21**, 165 (1994).
