# Supplementary material for: Crossover from lattice to plasmonic polarons of a spin-polarised electron gas in ferromagnetic EuO
Source: Nat Commun. 2018 Jun 13;9:2305. doi: 10.1038/s41467-018-04749-w (PMC5998015; doi:10.1038/s41467-018-04749-w)
Supplement: Supplementary file 2 — Peer Review File [file 41467_2018_4749_MOESM2_ESM.pdf]

Editorial Note: This manuscript has been previously reviewed at another journal that is not operating a transparent peer review scheme. This document only contains reviewer comments and rebuttal letters for versions considered at Nature Communications.

PEER REVIEW FILE

<u>Reviewers' comments</u>:

Reviewer #1 (Remarks to the Author):

In the revised manuscript and the reply to all reviewers, the authors well explain their achievement on plasmonic polaron beyond previous works. New figure 5 successfully demonstrates a distinct feature of plasmonic polaron that is not like the thought for conventional polaron. I also found adequate the author's comment to me about spin-polarization. As authors state, unwanted air sensitivity of EuO requiring in-situ measurement strongly limits a chance of spin-resolved AREPS at available facilities. Although I do not think that authors provide an evidence of spin-polarization of conduction band (magnetic properties in Fig. S7 is of Eu 4f electron), temperature dependence in Figure R2 presents an exchange-splitting nature of conduction band, effectively verifying a spin-polarization of conduction band. I thus recommend including this new data into supplementary information.
With such minor revisions, I think this work meets standards of publishing to Nature communications.

Reviewer #2:

None
